# 

**Published:** 1849-07

**Authors:** 


### CONTENTS.

### PART I.—ORIGINAL COMMUNICATIONS.

ART 1.—Fevers of summer and winter; their causes and treatment. An address delivered before the Wabash Central Medical Association. By CHARLES G. BALLARD, M. D., President of the Association, 93

ART. 2.—Observations pertaining to the Pathology and Treatment of Typhoid Fever. By W. MATTHEWS, M. D., 104

ART. 3.—Nitrate of Silver in Membranous Croup. By V. M. SATTERLEE, M. D. 108

ART. 4.—Letter from JOEL HENDRICKS, M. D. 109

ART. 5.—The Sectional Teachings of Medicine. By DANIEL STAHL, M. D., 111

ART. 6.—Coroner's Inquest—cause of death, doubtful. W. GOFF, 118

ART. 7.—Case of Nasal Polypus of fourteen years standing, cured by the local application of Peach Leaves. By E. W. H. BECK, M. D., of Delphi, Ind., 122

### PART II.—REVIEWS AND NOTICES OF NEW WORKS.

ART. 1—Epidemic Cholera—its History, Causes, Pathology and Treatment. By C. B. COVENTRY, M. D., 124

ART 2.—Obstetrics: the Science and Art. By CHARLES D. MEIGS, M. D., 131

ART. 3.—Anaesthesia, or the employment of Chloroform and Ether, in Surgery, Midwifery, etc. By J. G. SIMPSON, M. D., F. R. S. E., 1848, pp. 248, 8 vo. 133

### PART III.—SELECTIONS.

ART. 1.—The Heart Clot, 133

ART. 2.—Diphtherite, or Membranous Croup, treated by Ammonia and Oil. By T. L. OGIER, M. D. 144

ART. 3.—On Influenza and Ozone, 146

ART. 4.—On Muriate of Opium, 148

ART. 5.—Belladonna in the nocturnal incontinence of urine in children, 149

ART. 6.—Influence of cutting the hair on health, 149

### PART IV.—EDITORIALS.

ART. 1.—Dr. Edwards' Report—Patent Medicines, 151

ART. 2.—American Medical Association, 160

ART. 3.—Chicago Marine Hospital, 175

ART. 4.—Rush Medical College, 176

ART. 5.—U. S. Pharmacopœia, 177

ART. 6.—Cholera, 178

ART. 7.—Miscellaneous Medical Intelligence, 179

ADDENDUM—Herrick on Cholera, 181

